# Efficacy and safety of dupilumab in patients with severe chronic hand eczema with inadequate response or intolerance to alitretinoin: a randomized, double-blind, placebo-controlled phase IIb proof-of-concept study

**DOI:** 10.1093/bjd/ljad156

**Published:** 2023-05-12

**Authors:** Angelique N Voorberg, Esmé Kamphuis, Wietske A Christoffers, Marie L A Schuttelaar

**Affiliations:** Department of Dermatology, University of Groningen, University Medical Center Groningen, Groningen, the Netherlands; Department of Dermatology, University of Groningen, University Medical Center Groningen, Groningen, the Netherlands; Department of Dermatology, Isala Dermatologic Center, Zwolle, the Netherlands; Department of Dermatology, University of Groningen, University Medical Center Groningen, Groningen, the Netherlands

## Abstract

**Background:**

Effective treatment options for patients with chronic hand eczema (CHE) are scarce. Dupilumab is licensed for the treatment of moderate-to-severe atopic dermatitis and has shown promising results for the treatment of hand eczema in other studies.

**Objectives:**

To evaluate the efficacy and safety of dupilumab in adult patients with severe CHE (subtypes recurrent vesicular hand eczema or chronic fissured hand eczema) who have an inadequate response/intolerance to alitretinoin, or when alitretinoin is medically inadvisable.

**Methods:**

In this 16-week, randomized, double-blind, placebo-controlled proof-of-concept phase IIb trial, patients with severe CHE were randomized 2 : 1 to dupilumab 300 mg or placebo subcutaneously every 2 weeks. Patients visited the outpatient clinic at the initiation of the study drug, and every 4 weeks until 16 weeks of treatment. The primary endpoint was the proportion of patients achieving at least a 75% improvement on the Hand Eczema Severity Index score (HECSI-75) at week 16. Adverse events were monitored during each visit. The study was registered on ClinicalTrials.gov (identifier NCT04512339).

**Results:**

In total, 30 patients were randomized, and 29 patients received the assigned study drug (dupilumab *n* = 20, placebo *n* = 9). At week 16, more patients achieved HECSI-75 in the dupilumab group than in the placebo group {95% [95% confidence interval (CI) 73.1–99.7] vs. 33% [95% CI 9.0–69.1]}. Dupilumab also showed greater least square mean percentage change from baseline to week 16 in peak pruritus Numerical Rating Scale compared with placebo [−66.5 ± 10.7 (95% CI −88.6 to −44.5) vs. −25.3 ± 17.0 (95% CI −60.1–9.4)]. Adverse events were similar for the dupilumab and placebo groups and were mostly mild. There were no serious adverse events, nor did any of the adverse events lead to discontinuation of the study drug.

**Conclusions:**

Dupilumab was efficacious and well tolerated. Larger studies of longer duration are needed to provide more evidence on the ­efficacy of dupilumab in CHE. Moreover, larger studies could also enable comparisons between clinical subtypes or aetiological ­diagnoses.

## Graphical Abstract

**Graphical Abstract:**
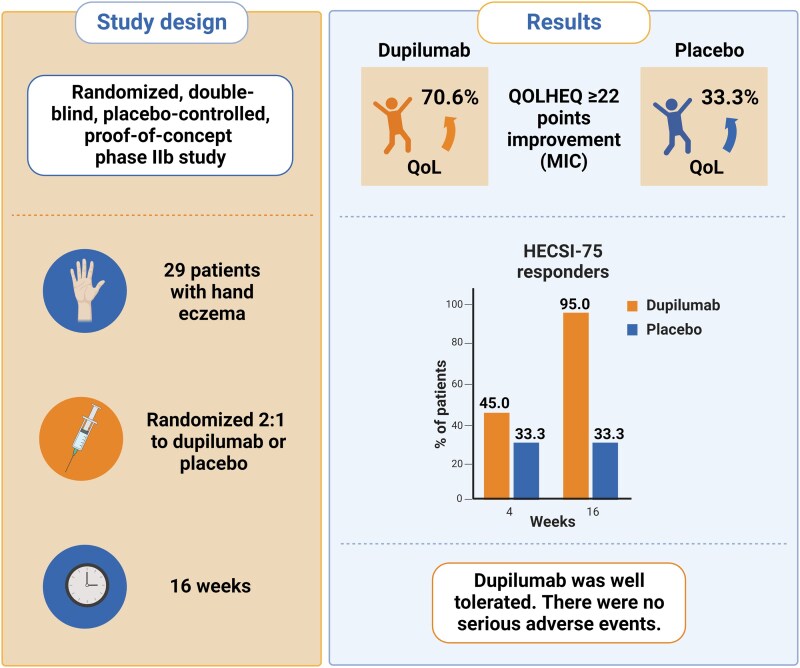

## Teaser

The findings in this 16-week proof-of-concept study demonstrated that dupilumab was an efficacious and well-tolerated treatment for patients with chronic hand eczema.

Linked Article: Brans *Br J Dermatol* 2023; 189:360–361.

Plain language summary available online

Author Video: https://youtu.be/Zwo5qUO4OGg

**What is already known about this topic?**

- Effective treatment options for severe chronic hand eczema (CHE) are scarce. This applies specifically to patients with severe CHE who have an inadequate response or intolerance to alitretinoin, or when alitretinoin is medically inadvisable.
- Dupilumab has shown promising results in observational studies for several subtypes of hand eczema (HE), including atopic and vesicular HE.

**What does this study add?**

- Treatment with dupilumab resulted in a larger proportion of patients with CHE achieving at least a 75% improvement on the Hand Eczema Severity Index after 16 weeks compared with placebo.
- Adverse events were similar in the dupilumab and placebo groups, and were mostly mild.
- The results of this proof-of-concept trial provide preliminary evidence of the efficacy and safety of dupilumab for severe CHE.

Even though hand eczema (HE) is common in the general population with a 1-year prevalence of up to 9.1%,^1^ the treatment options for HE are limited. Severe chronic HE (CHE) is often refractory to topical corticosteroids, resulting in the need for systemic treatment options. In most countries, alitretinoin is the only systemic treatment option licensed for all subtypes of severe CHE. However, post hoc analysis showed that alitretinoin is effective in hyperkeratotic HE, but is less effective in vesicular HE.^2^ Moreover, alitretinoin is registered for use up to 24 weeks and given the chronicity of HE, there is a need for additional treatment options.

Dupilumab, an interleukin (IL)-4/IL-13 inhibiting human monoclonal antibody licensed for the treatment of moderate-to-severe atopic dermatitis (AD), has shown promising results in observational studies for several subtypes of HE including atopic,^3^ hyperkeratotic^4^ and vesicular HE.^5,6^ Furthermore, in a recent published transcriptome study, the gene *IL4R*, which encodes the alpha chain of the IL-4 receptor, was found to be highly upregulated in lesional HE skin compared with healthy control skin.^7^ This suggests that the IL-4/IL-13 pathway might be involved in HE in patients without AD.

In this proof-of-concept phase IIb study, we report the efficacy and safety of dupilumab over 16 weeks of treatment in patients with severe CHE who have inadequate response or intolerance to alitretinoin, or when alitretinoin is medically inadvisable.

## Patients and methods

### Study design

This study (NCT04512339) was a 16-week, randomized, double-blind, placebo-controlled phase IIb monocentre clinical trial evaluating the efficacy and safety of dupilumab in adult patients with severe CHE. The trial was conducted from 18 August 2020, to 22 August 2022, at the Department of Dermatology of the University Medical Center Groningen, the Netherlands. The study, performed in accordance with the Declaration of Helsinki^8^ and Good Clinical Practice, was approved by the Dutch national competent authority (the Central Committee on Research Involving Human Subjects, reference number NL71585.042.19) and the local Ethical Review Board of the University Medical Center Groningen (METc 2019/500). All patients provided written informed consent before inclusion in the study.

### Patients

Patients were eligible if they were adults (aged 18–75 years) diagnosed with CHE (HE that lasts for more than 3 months or relapses twice or more often per year)^9,10^ who had inadequate response or intolerance to alitretinoin, or if they were patients for whom alitretinoin was medically inadvisable, and who had at least severe disease according to the photographic guide^11^ at the screening and baseline visits. In addition, patients were eligible for recruitment only if they had a clinical subtype of recurrent vesicular HE or chronic fissured HE according to the Danish guidelines for HE.^10^ Patients were required to have a washout period of at least 1 week for topical corticosteroids and topical calcineurin inhibitors, a washout period of at least 4 weeks for immunosuppressive and immunomodulating drugs (including methotrexate, alitretinoin, acitretin, azathioprine), with an exception for ciclosporin (washout period of 2 weeks) and prednisolone (washout period of 1 week). The washout for ultraviolet therapy was 4 weeks. Patients were required to be patch tested^12^ within 2 years prior to baseline. Patients with relevant contact sensitizations, for whom lack of avoidance of these allergens resulted in allergic contact dermatitis, were excluded. Patients who were exposed to irritant factors (occupational and nonoccupational) were eligible if avoidance of these factors was not feasible. Complete eligibility criteria can be found in Appendix S1 (see Supporting Information).

### Study procedures

Patients were randomized 2 : 1 to subcutaneous (s.c.) dupilumab injections (loading dose of 600 mg, followed by 300 mg every 2 weeks) or s.c. placebo injections. The loading dose was administered at the trial site, where patients were trained in the self-administration of the s.c. injections. Subsequent doses were performed by patients at home. Patients visited the outpatient clinic at screening, initiation of the study drug and every 4 weeks until 16 weeks of treatment had been completed.

Randomization was performed by the computer software ALEA (ALEA Clinical B.V., Abcoude, the Netherlands). The treatment allocation was blinded to all individuals, including the patient and investigators, using blinded and coded treatment kits. Only the data manager had immediate access to the list, which contained the deblinding codes in case of emergency.

Only the use of emollients was allowed, and no other cotreatment was allowed during the study period. Rescue therapy, defined as treatment with mometasone furoate ointment once daily for 1 week, could be initiated at investigator discretion (e.g. exacerbation of CHE). If patients received systemic corticosteroids or other systemic immunosuppressive or other systemic immunomodulating drugs during the trial, their trial participation was discontinued.

### Outcomes

The primary endpoint was the proportion of patients achieving at least 75% improvement on the Hand Eczema Severity Index (HECSI 75) score at week 16.^13^ Secondary key endpoints were achievement of at least 50% and 90% improvement on the HECSI (HECSI 50 and HECSI 90); mean (percentage) change in HECSI; proportion of patients achieving the minimal important change (MIC) of ≥ 41 on the HECSI; achievement of 0 (‘clear’) or 1 (‘almost clear’) on the Physician’s Global Assessment (PGA) (5-point scale instrument covering the following degrees of severity: clear, almost clear, mild, moderate and severe) with at least two steps improvement;^2^ mean (percentage) change on a modified version of the modified Total Lesion Symptom Score (mTLSS)^2^; mean (percentage) change in weekly average peak pruritus Numerical Rating Scale (NRS); proportion of patients achieving ≥ 4 points improvement on the weekly average peak pruritus NRS; mean (percentage) change in the Quality of Life in Hand Eczema Questionnaire (QOLHEQ);^14,15^ and the proportion of patients achieving the minimally important change MIC of ≥ 22 on the QOLHEQ.^16^ Other secondary endpoints were achievement of 0 (‘clear’) or 1 (‘almost clear’) on the photographic guide (5-point scale instrument covering the following degrees of severity: clear, almost clear, moderate, severe, very severe) with at least two steps improvement;^11^ mean (percentage) change in weekly average peak pain NRS; proportion of patients achieving ≥ 4 points improvement on the weekly average peak pain NRS; and achievement of 0 (‘clear’ or ‘almost clear’: at least 90% clearing of disease signs and symptoms’) or 1 (‘marked improvement’ and at least 75% clearing of disease signs and symptoms’) on the Patient’s Global Assessment.^2^ Quality of life was not only assessed using the QOLHEQ, but was also assessed using the Dermatology Life Quality Index (DLQI). Endpoints were the mean (percentage) change in the QOLHEQ subscales Symptoms, Emotions, Functioning, and Treatment and Prevention; proportion of patients achieving the smallest detectable change for the QOLHEQ subscales Symptoms, Emotions, Functioning, and Treatment and Prevention (symptoms ≥ 6 points; emotions ≥ 7 points, functioning ≥ 8 points; and treatment and prevention ≥ 5 points);^16^ and mean (percentage) change in DLQI.^17^ Furthermore, other secondary endpoints included percentage change in Work Productivity and Impairment Index (WPAI)^18^ and proportion of patients reporting no problems on the EuroQol Quality of Life 5-Dimension 5-Level (EQ-5D-5L) subdomains self-care, usual activities, pain/discomfort and anxiety/depression (further details on study procedures and outcomes are provided in Tables S1–S3; see Supporting Information).^19^

Safety outcomes included adverse events (AEs), laboratory tests and physical examination findings. AEs, including serious AEs (SAEs) and AEs of special interest (AESIs), were assessed and recorded at each visit during the 16 weeks of study treatment after initiation of the study drug. Routine laboratory assessments were performed every visit (Appendix S1).

### Statistical analysis

This proof-of-concept study was designed to provide preliminary evidence of efficacy in 30 patients, in order to provide the basis for a decision regarding further investigation of the efficacy of dupilumab in severe CHE. For binary outcomes, the χ^2^ test or likelihood-ratio test (in case of violations of the χ^2^ test) were used. Data collected prior to initiation of rescue medication and data prior to discontinuation of the study drug were used in the analyses. Data for patients after receipt of rescue therapy was not included in the analyses, and these patients were defined as nonresponders for binary endpoints after initiation of rescue medication. For the primary analysis of the binary outcomes, the last observation carried forward (LOCF) method was used to impute missing data. For all binary key outcomes, a sensitivity analysis was performed using the χ^2^ test of likelihood-ratio test and LOCF was used to impute missing data. In this sensitivity analysis, patients were considered as nonresponders after withdrawal from the trial owing to ineffectiveness and after use of rescue treatment. Additionally, sensitivity analyses for patients with or without (a history) of AD were performed. For continuous outcomes, least square means (LSMeans) were estimated using a mixed model for repeated measures and were used with an unstructured repeated covariance matrix, including an interaction between time and treatment. For the HECSI and the mTLSS analyses, an interaction between time and baseline assessment was also added. Missing data in the continuous outcomes were imputed using the mixed model for repeated measurements predicted values. Owing to the small sample size, confidence intervals (CIs) have been used rather than *P*-values for the LSMean estimates to infer size and direction of the treatment effect. Calculations were performed using IBM SPSS Statistics for Windows, version 28.0 (IBM, Armonk, NY, USA).

## Results

**Figure 1:**
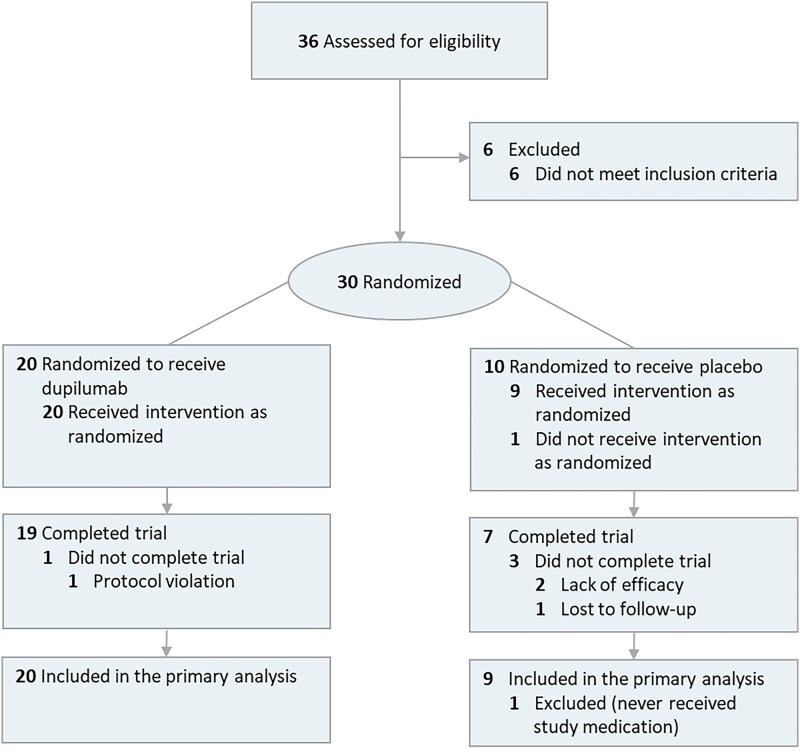
Flowchart showing patient disposition throughout the study up to week 16.

Between August 2020 and April 2022, 36 patients were screened, 30 of whom were randomized. Of these 30 patients, one patient did not receive the study treatment, as the patient did not have severe HE at baseline. This led to an analysis of 29 patients in total (dupilumab, *n* = 20; placebo, *n* = 9). In the dupilumab group, one patient discontinued trial participation between week 12 and week 16 owing to protocol-violating medication (see Figure 1). In the placebo group, two patients discontinued trial participation owing to ineffectiveness (one patient between week 4 and week 8 and one patient between week 8 and week 12) and one received rescue medication (between week 12 and week 16). Patient demographics and baseline characteristics were almost similar across treatment groups (see Table 1 and Table 2). Seventeen patients had irritant contact dermatitis as an HE contributing etiological subtype. A total of 16 patients were working in a high-risk occupation for HE. Almost all patients had at least one positive patch test reaction to an allergen. In the dupilumab group, five patients had a history of AD, one of whom had current AD. The median of number of systemic treatments in the past was 2.0 [interquartile range (IQR) 1.0–3.0].

**Table 1:** Baseline characteristics: patient demographics

Characteristics	All patients (n = 29)	Dupilumab (n = 20)	Placebo (n = 9)
Age (years), mean (SD)	43.9 (14.7)	44.5 (15.1)	42.8 (14.4)
Male	14 (48.3)	11 (55.0)	3 (33.3)
Female	15 (51.7)	9 (45.0)	6 (66.7)
Race			
White	27 (93.1)	18 (90.0)	9 (100.0)
Asian	1 (3.4)	1 (5.0)	0 (0.0)
Antillean	1 (3.4)	1 (5.0)	0 (0.0)
BMI (kg/m^2^), mean (SD)	27.4 (4.1)	27.6 (3.8)	27.0 (5.0)
Current smokers, *n* (%)	14 (48.3)	9 (45.0)	5 (55.6)
Ex-smokers, *n* (%)	9 (31.0)	8 (40.0)	1 (11.1)
Packyears, median [IQR]	7.0 [0.0-29.0]	7.5 [0.5-29.5]	7.0 [0.0-30.0]
Employed, *n* (%)	22 (75.9)	15 (75.0)	7 (77.8)
Working hours per week, median [IQR]	34.0 [26.5-40.0]	36.0 [28.0-45.0]	32.0 [25.0-36.0]
Working in a high-risk occupation for HE, *n* (%)^a^	16 (72.7)	10 (66.7)	6 (85.7)
Duration of disease in *y*, median [IQR]	10.0 [5.0-24.5]	13.5 [5.0-26.0]	5.0 [3.0-11.0]
Clinical subtype of HE, *n* (%)			
Chronic fissured	11 (37.9)	8 (40.0)	3 (33.3)
Recurrent vesicular	18 (62.1)	12 (60.0)	6 (66.7)
Etiological subtypes of HE			
Atopic HE	5 (17.2)	5 (25.0)	0 (0.0)
Irritant contact dermatitis, *n* (%)	17 (58.6)	10 (50.0)	7 (77.8)
Performing wet work, *n* (%)^a^	9 (31.0)	5 (25.0)	4 (44.4)
Protein contact dermatitis, *n* (%)	2 (6.9)	2 (10.0)	0 (0.0)
Contact sensitizations			
At least one positive patch test reaction to an allergen from the allergen groups, *n* (%)^b^	27 (93.1)	18 (90.0)	9 (100.0)
Metals	18 (62.1)	12 (60.0)	6 (66.7)
Preservatives	12 (41.4)	10 (50.0)	2 (22.2)
Fragrances	10 (34.5)	6 (30.0)	4 (44.4)
Rubbers	8 (27.6)	6 (30.0)	2 (22.2)
Dyes/colours	4 (13.8)	3 (15.0)	1 (11.1)
Topicals	8 (27.6)	7 (35.0)	1 (11.1)
Plastics	4 (13.8)	2 (10.0)	2 (22.2)
Other	3 (10.3)	2 (10.0)	1 (11.1)
Atopy, *n*/*n* (%)^c^	15 (51.7)	11 (55.0)	4 (44.4)
Atopic dermatitis			
Current atopic dermatitis, not needing medical attention	1 (3.4)	1 (5.0)	0 (0.0)
History of atopic dermatitis	5 (17.2)	5 (25.0)	0 (0.0)
Asthma	7 (21.4)	7 (35.0)	0 (0.0)
Allergic rhinitis	11 (37.9)	9 (45.0)	2 (22.2)
Allergic conjunctivitis	11 (37.9)	9 (45.0)	2 (22.2)
Food allergy	3 (10.3)	3 (15.0)	0 (0.0)
Total IgE level elevated (≥ 116 kU/L)	9 (31.0)	6 (30.0)	3 (33.3)
Number of systemic therapies in the past, median [IQR]^d^	2.0 [1.0-3.0]	2.4 [1.3-3.0]	2.0 [1.0-3.0]
Cyclosporine, *n* (%)	20 (69.0)	15 (75.0)	5 (55.6)
Prednisolone (short course), *n* (%)	22 (75.9)	16 (80.0)	6 (66.7)
Methotrexate, *n* (%)	7 (24.1)	5 (25.0)	2 (22.2)
Azathioprine, *n* (%)	8 (27.6)	5 (25.0)	3 (33.3)
Alitretinoin, *n* (%)	26 (89.7)	18 (90.0)	8 (88.9)
Reason for discontinuation			
Inadequate respons	18 (62.1)	11 (55.0)	7 (77.8)
Intolerance	4 (13.8)	4 (20.0)	0 (0.0)
Both (inadequate respons and intolerance	4 (13.8)	3 (15.0)	1 (11.1)
Medically inadvisable	3 (10.3)	2 (10.0)	1 (11.1)
Acitretin, *n* (%)	1 (3.4)	1 (5.0)	0 (0.0)
Mycophenolic acid or mycophenolate mofetil, *n* (%)	2 (6.9)	2 (10.0)	0 (0.0)
Triamcinolone acetonide injections, *n* (%)	1 (3.7)	1 (5.0)	0 (0.0)
Phototherapy in the past, *n* (%)	18 (62.1)	11 (55.0)	7 (77.8)
PUVA, *n* (%)	13 (44.8)	9 (45.0)	4 (44.4)
UVB, *n* (%)	6 (20.7)	3 (27.3)	3 (33.3)

AD, atopic dermatitis; BMI, body mass index; HE, hand eczema; IQR, interquartile range; PUVA, psoralen ultraviolet A; UVB, ultraviolet B. ^a^A total of 22 of 29 patients performed paid work at baseline. ^b^An overview of the allergen groups is provided in Table S2. ^c^Based on specific IgE inhalant allergens > 0.99. ^d^Number of systemic therapies minus prednisolone. Data are presented as *n* (%) unless otherwise stated.

**Table 2:** Baseline characteristics: severity and patient reported outcomes

Characteristics	All patients (*n* = 29)	Dupilumab (*n* = 20)	Placebo (*n* = 9)
HECSI score, mean (SD)	85.9 (42.8)	85.2 (45.6)	87.4 (37.5)
Severity photographic guide			
Severe	17 (59)	11 (55)	6 (67)
Very severe	12 (41)	9 (45)	3 (33)
PGA			
Severe	29 (100)	20 (100)	9 (100)
MTLSS score, mean (SD)	22.7 (5)	23.0 (6)	22.2 (4)
Peak NRS, median (IQR)			
Weekly average peak itch	6.6 (3.1–7.9)	6.5 (1.7–7.8)	6.7 (5.6–8.1)
Weekly average peak pain	6.0 (0.9–7.3)	6.3 (1.5–7.3)	4.9 (0.9–7.6)
QOLHEQ score, median (IQR)	61.0 (48.0–76.5)	59.5 (48.0–76.8)	70.0 (47.5–76.0)
QOLHEQ symptoms subscale score	19.0 (15.0–21.5)	19.5 (13.8–21.8)	18.0 (16.0–20.5)
QOLHEQ emotions subscale score	14.0 (9.0–21.0)	13.0 (6.5–19.5)	17.0 (11.5–22.5)
QOLHEQ functioning subscale score	16.0 (10.5–20.0)	16.0 (10.3–21.5)	17.0 (11.5–19.0)
QOLHEQ treatment and prevention subscale score	14.0 (8.5–17.0)	13.5 (7.5–17.0)	14.0 (9.5–17.0)
DLQI score, median (IQR)	11.0 (7.0–15.0)	11.5 (7.0–16.8)	10.0 (7.0–13.0)
WPAI scores, median (IQR)			
Absenteeism (%)	0 (0.0–0.69)	0 (0.0–0.69)	0 (0.0–0.0)
Presenteeism (%)	40 (22.5–57.5)	30 (5.0–55.0)	50 (40.0–70.0)
Overall work impairment (%)	40 (18.1–62.5)	30 (4.2–60.0)	50 (40.0–70.0)
Overall activity impairment (%)	50 (30.0–70.0)	30 (22.5–70.0)	50 (35.0–65.0)
EQ-5D-5L dimension			
Self-care: reporting ‘no problems’	14 (48)	8 (40)	6 (67)
Usual activities: reporting ‘no problems’	3 (10)	3 (15)	0 (0)
Pain/discomfort: reporting ‘no problems’	4 (14)	3 (15)	1 (11)
Anxiety/depression: reporting ‘no problems’	17 (59)	12 (60)	5 (56)

DLQI, Dermatology Life Quality Index; EQ-5D-5L, generic 5-dimension 5-level EuroQol scale; HECSI, Hand Eczema Severity Index; IQR, interquartile range; mTLSS, modified Total Lesion Symptom Score; NRS, Numerical Rating Scale; PGA, Physician’s Global Assessment; QOLHEQ, Quality of Life in Hand Eczema Questionnaire; WPAI, Work Productivity and Activity Impairment. Data are presented as *n* (%) unless otherwise stated.

### Primary outcome

**Figure 2:**
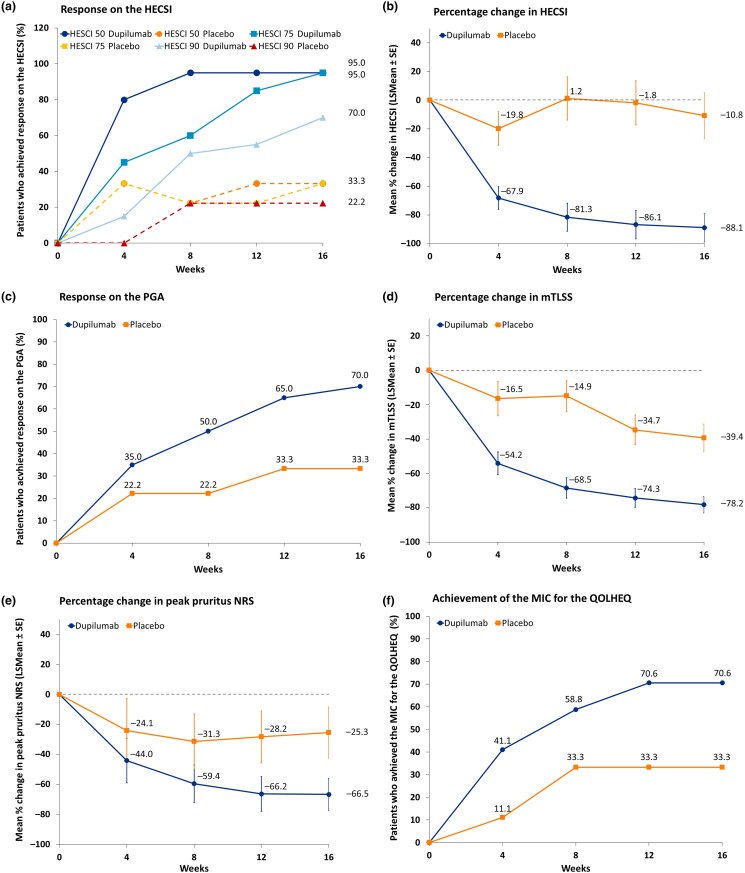
Key endpoints from baseline through week 16. (a) Primary endpoint: Hand Eczema Severity Index (HECSI). Lines show the proportion of patients who reached the primary endpoint of an improvement from baseline of at least 75% on the HECSI (HECSI 75) and the secondary endpoints of an improvement from baseline of at least 50% and 90% on the HECSI (HECSI 50 and HECSI 90). (b) Least square mean (LSMean) change in HECSI score, adjusted for baseline HECSI score. (c) Proportion responders on the Physician’s Global Assessment (PGA) (‘clear’ or ‘almost clear’ with at least two steps improvement). (d) LSMean percentage change in modified Total Lesion Symptom Score (mTLSS). Adjusted for baseline mTLSS score. (e) LSMean percentage change in Peak Pruritus Numerical Rating Scale (NRS) score. (f) Proportion of patients who achieved the minimally important change (MIC) for the Quality of Life in Hand Eczema Questionnaire (QOLHEQ) score. Binary endpoints (c and f) were analysed using the χ^2^ test or likelihood-ratio test and missing values were imputed using the last observation carried forward. Continuous endpoints (a, b, d and e) were analysed using a mixed model for repeated measurements and missing data were imputed using the mixed model for repeated measurements predicted values. SE, standard error.

At week 16, a larger proportion of patients in the dupilumab group achieved the primary endpoint of HECSI 75 compared with the placebo group [95.0% (95% CI 73.1–99.7) vs. 33.3% (95% CI 9.0–69.1)] (Figure 2a). The outcomes of the sensitivity analysis (Table S4; see Supporting Information) did not differ from the main analysis apart from one patient (who dropped out of the study between week 8 and week 12 owing to inefficacy) being considered as a nonresponder on all binary outcomes after withdrawal from the study. The sensitivity analyses for patients with a history of AD and those without a history of AD (Tables S5, S6; see Supporting Information) showed that slightly more patients without a history of AD achieved response on the binary key outcomes compared with patients who had a history of AD.

### Key secondary outcomes

The secondary endpoints of HECSI 50 and HECSI 90 were achieved by a larger proportion of patients in the dupilumab group compared with the placebo group at week 16 (HECSI 50: 95% vs. 33%; HECSI 90: 70% vs. 22%). The LSMean [ ± standard error] percentage change in HECSI from baseline to week 4 was −68.1 ± 7.9 (95% CI −84.3 to −52.0) in the dupilumab group and −19.8 ± 11.7 (95% CI −43.9–4.3) in the placebo group. The LSMean percentage change in HECSI from baseline to week 16 was −88.1 ± 10.1 (95% CI −109.6 to −68.1) in the dupilumab group and −10.8 ± 16.1 (95% CI −43.7–22.1) in the placebo group (Figure 2b). Response measured using the PGA at week 16 was achieved by 70% in the dupilumab group and 33% in the placebo group (Figure 2c). The LSMean percentage change in mTLSS from baseline to week 16 was −78.2 ± 4.8 (95% CI −88.2 to −68.1) in the dupilumab group and −39.4 ± 8.1 (95% CI −56.9 to −20.8) in the placebo group (Figure 2d). Dupilumab also showed greater LSMean percentage change improvement from baseline to week 16 in weekly average peak pruritus NRS than placebo [−66.5 ± 10.7 (95% CI −88.6 to −44.5) vs. −25.3 ± 17.0 (95% CI −60.1–9.4)] (Figure 2e). In both groups, patients reported improvement on the QOLHEQ compared with baseline. The proportion of patients achieving the MIC of 22 points^16^ for the QOLHEQ at week 16 was 71% compared in the dupilumab group with 33.3% in the placebo group (Figure 2f).

### Other secondary outcomes

All outcomes are presented in Table 3. Quality of life, assessed using the QOLHEQ and the DLQI, improved from baseline at all visits from week 4. For the WPAI, patients in the placebo group had an increase of 29.2% ± 7.3 (95% CI 13.3–45.2) in absenteeism at week 16 compared with a decrease of −5.4% ± 4.6 (95% CI −15.5–4.8) in the dupilumab group (Table 3 and Figure S1; see Supporting Information). In the dupilumab group, presenteeism and activity impairment improved from baseline, with more improvement for activity impairment in the dupilumab group compared with placebo at week 4 [−35.0 ± 6.2 (95% CI −47.4 to −22.3) vs. −10.0 ± 9.2 (95% CI −28.9–8.9)] and week 8 [−40.0 ± 6.1 (95% CI −52.6 to −27.4) vs. −12.7 ± 9.3 (95% CI −31.6–6.3)]. All generic 5-dimension 5-level EuroQol scale (EQ-5D-5L) dimensions except for anxiety/depression improved compared with baseline, with more patients compared with baseline reporting no problems for self-care (80%), usual activities (60%) and pain/discomfort (45%) (Table S3; see Supporting Information).

**Table 3:** Primary and secondary efficacy outcomes

Outcome	Week 4		Week 16	
	Dupilumab (*N* = 20)	Placebo (*N* = 9)	Dupilumab (*N* = 20)	Placebo (*N* = 9)
HECSI				
HECSI 50 response	16/20 (80)	3/9 (33)	19/20 (95)	3/9 (33)
HECSI 75 response (primary outcome)	9/20 (45)	3/9 (33)	19/20 (95)	3/9 (33)
HECSI 90 response	3/20 (15)	0/9 (0)	14/20 (70)	2/9 (22)
LSMean percentage change from baseline ± SE (95% CI)	−68.1 ± 7.9 (−84.3 to −52.0)	−19.8 ± 11.7 (−43.9–4.3)	−88.1 ± 10.1 (−109.6 to −68.1)	−10.8 ± 16.1 (−43.7–22.1)
LSMean change from baseline ± SE (95% CI)^a^	−53.0 ± 8.6 (−70.7 to −35.3)	−8.7 ± 12.8 (−35.1–17.7)	−73.2 ± 11.2 (−96.6 to −49.7)	9.5 ± 17.4 (−26.6–45.7)
≥ 41 points improvement from baseline	13/15 (87)	3/8 (38)	14/15 (93)	2/8 (25)
mTLSS				
LSMean percentage change from baseline ± SE (95% CI)^b^	−54.2 ± 6.6 (−67.9 to −40.5)	−16.5 ± 9.9 (−36.9 to 3.8)	−78.2 ± 4.8 (−88.2 to −68.1)	−39.4 ± 8.1 (−56.1 to −22.7)
LSMean change from baseline ± SE (95% CI)^b^	−11.9 ± 1.4 (−14.8 to −9.0)	−4.4 ± 2.1 (−8.7 to −0.1)	−17.3 ± 1.2 (−19.9 to −14.8)	−7.9 ± 2.1 (−12.2 to −3.7)
PG				
Responders (‘clear’ or ‘almost clear’ and ≥ 2 points improvement)	7/20 (35)	2/9 (22)	14/20 (70)	3/9 (33)
PGA				
Responders (‘clear’ or ‘almost clear’ and ≥ 2 points improvement)	7/20 (35)	2/9 (22)	14/20 (70)	3/9 (33)
Peak pruritus NRS				
LSMean percentage change from baseline ± SE (95% CI)	−44.0 ± 14.8 (−74.4 to −13.5)	−24.1 ± 21.5 (−68.4 to 20.1)	−66.5 ± 10.7 (−88.6 to −44.5)	−25.3 ± 17.0 (−60.1 to 9.4)
LSMean change from baseline ± SE (95% CI)	−2.4 ± 0.5 (−3.4 to −1.3)	−1.9 ± 0.7 (−3.4 to −0.4)	−3.3 ± 0.6 (4.5 to −2.1)	−2.4 ± 0.9 (−4.4 to −0.6)
≥ 4 points improvement from baseline	6/13 (46)	2/8 (25)	10/13 (77)	3/8 (38)
Peak pain NRS				
LSMean percentage change from baseline ± SE (95% CI)	−70.4 ± 65.7 (−206.3–65.6)	145.3 ± 95.8 (−52.8–343.5)	−79.8 ± 10.6 (−101.9 to −57.7)	−39.3 ± 17.3 (−74.8 to −3.8)
LSMean change from baseline ± SE (95% CI)	−3.3 ± 0.7 (−4.7 to −1.8)	−0.3 ± 1.0 (−2.4–1.8)	−3.8 ± 0.7 (−5.1 to −2.4)	−2.4 ± 1.0 (−4.5 to −0.3)
≥ 4 points improvement from baseline	11/15 (73)	2/6 (33)	12/15 (80)	3/6 (50)
PaGA				
Responders (‘clear’ or ‘almost clear’)	5/20 (25)	0/9 (0)	10/20 (50)	2/9 (22)
QOLHEQ				
LSMean percentage change from baseline ± SE (95% CI)	−28.5 ± 6.0 (−40.9 to −16.2)	−21.9 ± 9.0 (40.3 to −3.5)	−56.5 ± 5.9 (−68.6 to −44.3)	−38.2 ± 9.9 (−58.4 to −18.0)
LSMean change from baseline ± SE (95% CI)	−19.9 ± 4.3 (−28.7 to −11.1)	−13.1 ± 6.4 (−26.2–0.0)	−32.3 ± 4.7 (−41.8 to −22.7)	−23.9 ± 7.4 (−38.9 to −8.9)
≥22 points improvement from baseline	7/17 (41)	1/9 (11)	12/17 (71)	3/9 (33)
DLQI				
LSMean percentage change from baseline ± SE (95% CI)	−63.6 ± 10.7 (−85.6 to −41.7)	−32.8 ± 15.5 (−64.7 to −1.0)	−73.9 ± 6.2 (−86.7 to −61.1)	−64.7 ± 10.2 (−85.8 to −43.6)
LSMean change from baseline ± SE (95% CI)	−7.2 ± 1.5 (−10.3 to −4.1)	−3.2 ± 2.3 (7.9–1.4)	−8.6 ± 1.4 (11.4 to −5.7)	−5.4 ± 2.2 (−9.9 to −0.9)
≥ 4 points improvement from baseline	15/18 (83)	5/9 (56)	15/18 (83)	7/9 (78)
WPAI				
Absenteeism: LSMean percentage change from baseline ± SE (95% CI)	−0.1 ± 4.6 (−9.7–9.6)	4.8 ± 6.4 (−8.8–18.4)	−5.4 ± 4.6 (−15.5–4.8)	29.2 ± 7.3 (13.3–45.2)
Presenteeism: LSMean percentage change from baseline ± SE (95% CI)	−19.1 ± 5.5 (−30.6 to −7.6)	−18.6 ± 7.3 (−33.9 to −3.2)	−19.3 ± 6.9 (−34.0 to −4.7)	−22.1 ± 10.3 (−43.8 to −0.5)
Overall work impairment: LSMean percentage change from baseline ± SE (95% CI)	−14.8 ± 6.4 (−28.2 to −1.4)	−11.1 ± 9.2 (−30.3–8.1)	−19.0 ± 6.1 (−29.4–0.6)	−1.8 ± 13.0 (−23.3–23.9)
Activity impairment: LSMean percentage change from baseline ± SE (95% CI)	−35.0 ± 6.2 (−47.7 to −22.3)	−10.0 ± 9.2 (−28.9–8.9)	−29.7 ± 6.1 (−42.7 to −18.3)	−16.1 ± 10.5 (−35.0–4.9)

CI, confidence interval; DLQI, Dermatology Life; HECSI, Hand Eczema Severity Index; LS, least square; mTLSS, modified Total Lesion Symptom Score; NRS, Numerical Rating Scale; PG, photographic guide; PGA, Physician’s Global Assessment; QOLHEQ, Quality of Life in Hand Eczema Questionnaire; SE, standard error; WPAI, Work Productivity and Activity Impairment. Binary endpoints were analysed using the χ^2^ test or likelihood-ratio test and missing values, e.g. data from patients after withdrawal from the study, were imputed using the last observation carried forward. Continuous endpoints were analysed using a mixed model for repeated measurements and missing data were imputed using the mixed model for repeated measurements predicted values. Patients with < 4 points on the baseline peak pruritus NRS were excluded from the ≥ 4 points improvement on peak pruritus NRS analysis. Patients with < 4 points on baseline peak pain NRS were excluded from the ≥ 4 points improvement on peak pain NRS analysis. Patients with < 22 points on baseline total QOLHEQ scores were excluded from the ≥ 22 points improvement on total QOLHEQ analysis. Patients with < 4 points on baseline DLQI were excluded from the ≥ 4 points improvement on DLQI analysis. For the WPAI, questions regarding absenteeism, presenteeism and overall work impairment were answered only by employed patients (13/20 patients in the dupilumab group, 7/9 patients in the placebo group). The question regarding activity impairment was answered by all patients. Patients who scored 0.0% on baseline scores for absenteeism, presenteeism, overall work impairment or activity impairment were not included in this analysis. ^a^Adjusted for baseline HECSI score. ^b^Adjusted for baseline mTLSS. Data are provided as *n*/*N*(%) unless otherwise stated.

### Safety

All AEs that occurred are presented in Table 4. Dupilumab was well tolerated and there was no notable difference in the proportions of patients reporting AEs in the dupilumab group compared with the placebo group. No SAEs or AESIs occurred during this trial, and in both groups AEs did not lead to discontinuation of the study drug. The most frequent adverse event was ocular pruritus [3 of 20 (15%) in the dupilumab group, 0 of 9 (0%) in the placebo group], which occurred without any other subjective symptoms or clinical signs. Conjunctivitis did not occur during this study. Muscle or joint pain occurred in two patients (10%) in the dupilumab group and in none of the patients (0%) in the placebo group. The proportion of patients with eosinophilia did not increase after initiation of dupilumab. No clinically significant abnormalities were observed in other laboratory parameters.

**Table 4:** Overview of adverse events

Adverse event	Dupilumab (*N* = 20)	Placebo (*N* = 9)
Any adverse event, *n*	13	3
Patients with adverse events	11 (55)	3 (33)
Ocular pruritus^a^	3 (15)	0 (0)
Oedema	2 (10)	0 (0)
Muscle or joint pain	2 (10)	0 (0)
Dry eyes^b^	1 (5)	1 (11)
Panaritium	0 (0)	1 (11)
Localized redness of the conjunctivae^c^	0 (0)	1 (11)
Fatigue	1 (5)	0 (0)
Erysipelas	1 (5)	0 (0)
Facial redness	1 (5)	0 (0)
Gastrointestinal complaints	1 (5)	0 (0)
Prostate carcinoma	1 (5)^d^	0 (0)
Adverse events of special interest, *n*		
Conjunctivitis	0 (0)	0 (0)
Serious adverse events, *n*	0	0
Adverse events leading to discontinuation of study drug	0 (0)	0 (0)
Eosinophilia (≥ 0.45 × 10^9^ L^−1^) per visit^e^		
Baseline	2 (10)	1 (11)
Week 4	1 (5)	0 (0)
Week 8	0 (0)	0 (0)
Week 12	1 (5)	0 (0)
Week 16	1 (5)	1 (11)

^a^No other subjective symptoms or clinical signs. Artificial tears and antihistamine eyedrops were prescribed. ^b^No other subjective symptoms or clinical signs. Artificial tears were prescribed. ^c^No other subjective symptoms or clinical signs. ^d^Symptoms were pre-existent to the initiation of the study drug. ^e^All cases of eosinophilia were ≥0.45 × 10^9^ L^−1^ and ≤ 0.65 × 10^9^ L^−1^. *n*, number. Data are presented as *n* (%) unless otherwise stated.

## Discussion

The findings in this 16-week proof-of-concept study demonstrated that dupilumab was an efficacious and well-tolerated treatment for patients with CHE. Dupilumab showed greater improvement compared with placebo on the primary outcome (HECSI 75) and secondary end points, including the mTLSS and peak pruritus NRS.

In AD, dupilumab has already shown long-term effectiveness and long-term safety in an open-label extension study^20,21^ and in a daily practice drug survival study.^22^ The effect of dupilumab on CHE in patients with AD has been studied in a prospective, observational, daily practice study.^3^ In that study, 62.5% of the patients reached HECSI 75 at week 16, which increased to 87.1% after 52 weeks of treatment. In the current study, 95% (19 of 20) of the patients reached HECSI 75 after 16 weeks of dupilumab treatment and the majority of patients had no history of AD, indicating that dupilumab is not only effective for AD, but also for HE.

Our study showed that 70% (14 of 20) of the patients achieved ‘clear’ or ‘almost clear’ and ≥ 2 points improvement on the PGA after 16 weeks of dupilumab treatment. In a randomized, placebo-controlled multicentre trial, 47.7% of the patients treated with alitretinoin 30 mg achieved ‘clear’ or ‘almost clear’ after 16 weeks of treatment on a 5-point Investigator’s Global Assessment.^2^ In a phase IIb randomized, vehicle-controlled, multicentre, dose-ranging trial, 37.7% of the patients receiving the topical pan-Janus kinase (JAK) inhibitor delgocitinib 20 mg g^−1^ achieved ‘clear’ or ‘almost clear’ and ≥ 2 points improvement on a 5-point Investigator’s Global Assessment after 16 weeks.^23^ Trials with larger sample sizes, in addition to head-to-head trials, are needed to evaluate whether dupilumab is more effective than other treatment options for HE.

Itch is considered as one of the most common and burdensome symptoms experienced by patients with HE.^24^ In addition, itch has a negative impact on their quality of life. In our study, dupilumab showed an improvement from baseline to week 16 in peak pruritus NRS. The proportion of patients treated with dupilumab achieving the MIC of 22 points for the QOLHEQ at week 16 was 71% (12 of 17 patients) in our study, which is comparable to a daily practice study on the effect of dupilumab on HE in patients with AD, in which 77.2% achieved the MIC after 52 weeks.^3^

The WPAI, which assesses the impact of disease on the ability to work and to perform regular activities,^18^ was included as an outcome in this study as HE can lead to sickness absenteeism, presenteeism and eventually even to job loss or change of profession.^25^ In our study, patients in the placebo group experienced an increase in absenteeism at week 16 compared with the dupilumab group. Furthermore, LSMean percentage change in presenteeism and activity impairment decreased from baseline to week 16 in the dupilumab group. Long-term studies with a larger sample size are needed to draw firm conclusions on the effect of dupilumab on work and activity impairment.

The most common AEs in the dupilumab group was unspecified eye-related complaints, e.g. ocular pruritus without other subjective symptoms or clinical signs. There were no reported or observed cases of conjunctivitis, which is in line with other non-AD trials for dupilumab, such as asthma^26^ and chronic rhinosinusitis with nasal polyposis.^27,28^

In our study, a high placebo effect of 33% for the primary outcome of HECSI 75 was observed. Several factors may have contributed to this high placebo effect. First of all, in a smaller sample size, such as this study, a higher placebo effect becomes more likely.^29^ Furthermore, in a randomized controlled study, it was found that patients with AD show a higher placebo effect and maintain this placebo effect longer than persons with healthy skin.^30^ This might also be the case in HE. In contrast to our study, a rather low placebo effect of 8.0% was observed at week 16 in the phase IIb trial for the topical pan-JAK inhibitor delgocitinib in patients with moderate-to-severe CHE.^23^ However, the magnitude of the placebo effect might also depend on the treatment vehicle (injections vs. topical treatment) and the included range of disease severity. The small sample size is the main limitation of this study. Another limitation is the relatively short duration of our study, which limits the ability to assess improvement in quality of life, anxiety/depression and WPAI. Finally, in this study only two morphological HE subtypes were eligible for recruitment, i.e. recurrent vesicular HE or chronic fissured HE. Therefore, we cannot draw any conclusions regarding the efficacy of dupilumab in other HE subtypes based on this study.

In summary, dupilumab treatment was effective and well tolerated. Larger studies, preferably with longer duration, are needed to provide more evidence on the efficacy and safety of dupilumab in HE. Moreover, larger studies could also enable comparisons between CHE subtypes, for example irritant contact dermatitis or a history of AD.

## Supplementary Material

ljad156_Supplementary_Data

## Data Availability

The data that support the findings of this study are available upon reasonable request.
