# Correction to: Efficacy and safety of dupilumab in patients with severe chronic hand eczema with inadequate response or intolerance to alitretinoin: a randomized, double-blind, placebo-controlled phase IIb proof-of-concept study

**DOI:** 10.1093/bjd/ljad482

**Published:** 2023-12-20

This is a correction to: Angelique N Voorberg, Esmé Kamphuis, Wietske A Christoffers, Marie L A Schuttelaar, Efficacy and safety of dupilumab in patients with severe chronic hand eczema with inadequate response or intolerance to alitretinoin: a randomized, double-blind, placebo-controlled phase IIb proof-of-concept study, *British Journal of Dermatology*, Volume 189, Issue 4, October 2023, Pages 400–409, https://doi.org/10.1093/bjd/ljad156

In the originally published version of this manuscript there was an error in the layout of Table 1. Table 1 should read:

**Table 1:** Baseline characteristics: patient demographics

Characteristics	All patients (n = 29)	Dupilumab (n = 20)	Placebo (n = 9)
Age (years), mean (SD)	43.9 (14.7)	44.5 (15.1)	42.8 (14.4)
Male	14 (48.3)	11 (55.0)	3 (33.3)
Female	15 (51.7)	9 (45.0)	6 (66.7)
Race			
White	27 (93.1)	18 (90.0)	9 (100.0)
Asian	1 (3.4)	1 (5.0)	0 (0.0)
Antillean	1 (3.4)	1 (5.0)	0 (0.0)
BMI (kg/m^2^), mean (SD)	27.4 (4.1)	27.6 (3.8)	27.0 (5.0)
Current smokers, *n* (%)	14 (48.3)	9 (45.0)	5 (55.6)
Ex-smokers, *n* (%)	9 (31.0)	8 (40.0)	1 (11.1)
Packyears, median [IQR]	7.0 [0.0-29.0]	7.5 [0.5-29.5]	7.0 [0.0-30.0]
Employed, *n* (%)	22 (75.9)	15 (75.0)	7 (77.8)
Working hours per week, median [IQR]	34.0 [26.5-40.0]	36.0 [28.0-45.0]	32.0 [25.0-36.0]
Working in a high-risk occupation for HE, *n* (%)^a^	16 (72.7)	10 (66.7)	6 (85.7)
Duration of disease in *y*, median [IQR]	10.0 [5.0-24.5]	13.5 [5.0-26.0]	5.0 [3.0-11.0]
Clinical subtype of HE, *n* (%)			
Chronic fissured	11 (37.9)	8 (40.0)	3 (33.3)
Recurrent vesicular	18 (62.1)	12 (60.0)	6 (66.7)
Etiological subtypes of HE			
Atopic HE	5 (17.2)	5 (25.0)	0 (0.0)
Irritant contact dermatitis, *n* (%)	17 (58.6)	10 (50.0)	7 (77.8)
Performing wet work, *n* (%)^a^	9 (31.0)	5 (25.0)	4 (44.4)
Protein contact dermatitis, *n* (%)	2 (6.9)	2 (10.0)	0 (0.0)
Contact sensitizations			
At least one positive patch test reaction to an allergen from the allergen groups, *n* (%)^b^	27 (93.1)	18 (90.0)	9 (100.0)
Metals	18 (62.1)	12 (60.0)	6 (66.7)
Preservatives	12 (41.4)	10 (50.0)	2 (22.2)
Fragrances	10 (34.5)	6 (30.0)	4 (44.4)
Rubbers	8 (27.6)	6 (30.0)	2 (22.2)
Dyes/colours	4 (13.8)	3 (15.0)	1 (11.1)
Topicals	8 (27.6)	7 (35.0)	1 (11.1)
Plastics	4 (13.8)	2 (10.0)	2 (22.2)
Other	3 (10.3)	2 (10.0)	1 (11.1)
Atopy, *n*/*n* (%)^c^	15 (51.7)	11 (55.0)	4 (44.4)
Atopic dermatitis			
Current atopic dermatitis, not needing medical attention	1 (3.4)	1 (5.0)	0 (0.0)
History of atopic dermatitis	5 (17.2)	5 (25.0)	0 (0.0)
Asthma	7 (21.4)	7 (35.0)	0 (0.0)
Allergic rhinitis	11 (37.9)	9 (45.0)	2 (22.2)
Allergic conjunctivitis	11 (37.9)	9 (45.0)	2 (22.2)
Food allergy	3 (10.3)	3 (15.0)	0 (0.0)
Total IgE level elevated (≥ 116 kU/L)	9 (31.0)	6 (30.0)	3 (33.3)
Number of systemic therapies in the past, median [IQR]^d^	2.0 [1.0-3.0]	2.4 [1.3-3.0]	2.0 [1.0-3.0]
Cyclosporine, *n* (%)	20 (69.0)	15 (75.0)	5 (55.6)
Prednisolone (short course), *n* (%)	22 (75.9)	16 (80.0)	6 (66.7)
Methotrexate, *n* (%)	7 (24.1)	5 (25.0)	2 (22.2)
Azathioprine, *n* (%)	8 (27.6)	5 (25.0)	3 (33.3)
Alitretinoin, *n* (%)	26 (89.7)	18 (90.0)	8 (88.9)
Reason for discontinuation			
Inadequate respons	18 (62.1)	11 (55.0)	7 (77.8)
Intolerance	4 (13.8)	4 (20.0)	0 (0.0)
Both (inadequate respons and intolerance	4 (13.8)	3 (15.0)	1 (11.1)
Medically inadvisable	3 (10.3)	2 (10.0)	1 (11.1)
Acitretin, *n* (%)	1 (3.4)	1 (5.0)	0 (0.0)
Mycophenolic acid or mycophenolate mofetil, *n* (%)	2 (6.9)	2 (10.0)	0 (0.0)
Triamcinolone acetonide injections, *n* (%)	1 (3.7)	1 (5.0)	0 (0.0)
Phototherapy in the past, *n* (%)	18 (62.1)	11 (55.0)	7 (77.8)
PUVA, *n* (%)	13 (44.8)	9 (45.0)	4 (44.4)
UVB, *n* (%)	6 (20.7)	3 (27.3)	3 (33.3)

AD, atopic dermatitis; BMI, body mass index; HE, hand eczema; IQR, interquartile range; PUVA, psoralen ultraviolet A; UVB, ultraviolet B. ^a^A total of 22 of 29 patients performed paid work at baseline. ^b^An overview of the allergen groups is provided in Table S2. ^c^Based on specific IgE inhalant allergens > 0.99. ^d^Number of systemic therapies minus prednisolone. Data are presented as *n* (%) unless otherwise stated.

instead of:

**Table 1:** Baseline characteristics: patient demographics

Characteristics	All patients (*n* = 29)	Dupilumab (*n* = 20)	Placebo (*n* = 9)
Age (years), mean (SD)	43.9 (14.7)	44.5 (15.1)	42.8 (14.4)
Sex			
Male	14 (48)	11 (55)	3 (33)
Female	15 (52)	9 (45)	6 (67)
Race/ethnicity			
White	27 (94)	18 (90)	9 (100)
Asian	1 (3)	1 (5)	0 (0)
Antillean	1 (3)	1 (5)	0 (0)
BMI (kg m^−^²), mean (SD)	27.4 (4.1)	27.6 (3.8)	27.0 (5.0)
Current smokers	14 (48)	9 (45)	5 (56)
Ex-smokers	9 (31)	8 (40)	1 (11)
Pack-years, median (IQR)	7.0 (0.0–29.0)	7.5 (0.5–29.5)	7.0 (0.0–30.0)
Employed	22 (76)	15 (75)	7 (78)
Working hours per week, median (IQR)	34.0 (26.5–40.0)	36.0 (28.0–45.0)	32.0 (25.0–36.0)
Working in a high-risk occupation for HE^a^	16 (73)	10 (67)	6 (86)
Duration of disease in years, median (IQR)	10.0 (5.0–24.5)	13.5 (5.0–26.0)	5.0 (3.0–11.0)
Clinical subtype of HE			
Chronic fissured	11 (38)	8 (40)	3 (33)
Recurrent vesicular	18 (62)	12 (60)	6 (67)
Aetiological subtypes of HE			
Atopic HE	5 (17)	5 (25)	0 (0)
Irritant contact dermatitis	17 (59)	10 (50)	7 (78)
Performing wet work^a^	9 (31)	5 (25)	4 (44)
Protein contact dermatitis	2 (7)	2 (10)	0 (0)
Contact sensitizations			
At least one positive patch test reaction to an allergen from the allergen groups, *n* (%)^b^	27 (93)	18 (90)	9 (100)
Metals	18 (62)	12 (60)	6 (67)
Preservatives	12 (41)	10 (50)	2 (22)
Fragrances	10 (35)	6 (30)	4 (44)
Rubbers	8 (28)	6 (30)	2 (22)
Dyes/colours	4 (14)	3 (15)	1 (11)
Topicals	8 (28)	7 (35)	1 (11)
Plastics	4 (14)	2 (10)	2 (22)
Other	3 (10)	2 (10)	1 (11)
Atopy^c^			
Atopic dermatitis	15 (52)	11 (55)	4 (44)
Current AD, not needing medical attention	1 (3)	1 (5)	0 (0)
History of atopic dermatitis	5 (17)	5 (25)	0 (0)
Asthma	7 (21)	7 (35)	0 (0)
Allergic rhinitis	11 (38)	9 (45)	2 (22)
Allergic conjunctivitis	11 (38)	9 (45)	2 (22)
Food allergy	3 (10)	3 (15)	0 (0)
Total IgE level elevated (≥ 116 kU L^−1^)	9 (31)	6 (30)	3 (33)
Number of systemic therapies in the past, median (IQR)^d^	2.0 (1.0–3.0)	2.4 (1.3–3.0)	2.0 (1.0–3.0)
Ciclosporin	20 (69)	15 (75)	5 (56)
Prednisolone (short course)	22 (76)	16 (80)	6 (67)
Methotrexate	7 (24)	5 (25)	2 (22)
Azathioprine	8 (28)	5 (25)	3 (33)
Alitretinoin	26 (90)	18 (90)	8 (89)
Reason for discontinuation			
Inadequate response	18 (62)	11 (55)	7 (78)
Intolerance	4 (14)	4 (20)	0 (0)
Both (inadequate response and intolerance)	4 (14)	3 (15)	1 (11)
Medically inadvisable	3 (10)	2 (10)	1 (11)
Acitretin	1 (3)	1 (5)	0 (0)
Mycophenolic acid or mycophenolate mofetil	2 (7)	2 (10)	0 (0)
Triamcinolone acetonide injections	1 (4)	1 (5)	0 (0)
Phototherapy in the past	18 (62)	11 (55)	7 (78)
PUVA	13 (45)	9 (45)	4 (44)
UVB	6 (21)	3 (27)	3 (33)

AD, atopic dermatitis; BMI, body mass index; HE, hand eczema; IQR, interquartile range; PUVA, psoralen ultraviolet A; UVB, ultraviolet B. ^a^A total of 22 of 29 patients performed paid work at baseline. ^b^An overview of the allergen groups is provided in Table S2. ^c^Based on specific IgE inhalant allergens > 0.99. ^d^Number of systemic therapies minus prednisolone. Data are presented as *n* (%) unless otherwise stated.

In section **Results,** first paragraph, the 7th sentence should read: “Seventeen patients had irritant contact dermatitis as an HE contributing etiological subtype.” instead of: “Overall, 17 patients had International Classification of Diseases codes with HE as a contributing aetiological subtype.”

These errors have been emended in the article online only.

