# Supplementary material for: Efficacy and safety of dupilumab in patients with severe chronic hand eczema with inadequate response or intolerance to alitretinoin: a randomized, double-blind, placebo-controlled phase IIb proof-of-concept study
Source: Br J Dermatol. 2023 May 12;189(4):400–9. doi: 10.1093/bjd/ljad156 (PMC13077220; doi:10.1093/bjd/ljad156)
Supplement: ljad156_Supplementary_Data [file ljad156_supplementary_data.docx]

**Supporting Information**

**Efficacy and safety of dupilumab in patients with severe chronic hand eczema with inadequate response or intolerance to alitretinoin: a randomized, double-blind, placebo-controlled phase IIb proof-of-concept study.**

Angelique N. Voorberg^1^, Esmé Kamphuis^1^, Wietske A. Christoffers², Marie L.A. Schuttelaar^1^

^1^Department of Dermatology, University of Groningen, University Medical Center Groningen, Groningen, The Netherlands.

²Department of Dermatology, Isala Dermatologic Center, Zwolle, The Netherlands.

**Appendix S1. Supplemental methods**

*Eligibility criteria*

Inclusion criteria

In order to be eligible to participate in this study, a subject must meet all of the following criteria:

- Age ≥ 18 years and ≤ 75 years.
- Severe or very severe chronic hand eczema (HE) as defined by the validated photographic guide^1^.
- Recurrent vesicular HE or chronic fissured HE according to the Danish guidelines for HE^2^.
- An inadequate response to topical corticosteroids within 6 months before screening.
- A history of inadequate response to alitretinoin treatment, intolerance of alitretinoin or contraindications for alitretinoin.
- Having applied standard skin care, including emollients and barrier protection as appropriate, without significant improvement.
- Women of childbearing potential are required to use a highly effective (failure rate of <1% per year when used consistently and correctly) method of birth control, prior to receiving study intervention, during the study and for at least 10 weeks after receiving the last administration of study intervention. E.g., established use of oral, injected or implanted hormonal methods of contraception; placement of an intrauterine device or intrauterine system; barrier methods: condom or occlusive cap (diaphragm or cervical/vault caps) plus spermicidal foam/gel/film/cream/suppository (if available in their locale); male partner sterilization (the vasectomized partner should be the sole partner for that participant); true abstinence (when this is in line with the preferred and usual lifestyle of the participant). NOTE: If a female participant’s childbearing potential changes after start of the study (e.g., a woman who is not heterosexually active becomes active, a premenarchal woman experiences menarche), she must begin practicing a highly effective method of birth control, as described above.
- A woman of childbearing potential must have a negative serum or urine pregnancy test (β-human chorionic gonadotropin [β-hCG]) at screening prior to administration of study intervention.
- Agree not to receive a live virus or live bacterial vaccination during the study, or within 12 weeks after the last administration of study intervention.
- Agree not to receive a BCG vaccination during the study, or within 12 months after the last administration of study intervention.
- Be willing and able to adhere to the prohibitions and restrictions specified in this protocol.
- Must sign an informed consent form (ICF) indicating that he or she understands the purpose of and procedures required for the study, and is willing to participate in the study.

Exclusion criteria

A potential subject who meets any of the following criteria will be excluded from participation in this study:

- Other clinical subtypes of HE, e.g. hyperkeratotic HE, as defined by the Danish Contact Dermatitis Group^2^.
- Treatment with topical corticosteroids or topical calcineurin inhibitors within the previous week, treatment with immunosuppressive and immunomodulating drugs (including methotrexate, alitretinoin, acitretin, azathioprine) within the previous 4 weeks, with an exception for cyclosporine (wash-out period of two weeks) and prednisolone (wash-out period of one week). The wash-out for UV therapy was four weeks.
- Patients with atopic dermatitis (AD) on other parts of the body than the hands, which needs medical attention.
- Patients for whom relevant contact allergens for HE have been identified through patch testing.
- Psoriasis of the hands.
- Active bacterial, fungal, or viral infection of the hands.
- Pregnant/lactating or planning to become pregnant during the study period.
- Current malignancy (other than successfully treated non-metastatic cutaneous squamous cell or basal cell carcinoma and/or localized carcinoma in situ of the cervix).
- Participant has known allergies, hypersensitivity, or intolerance to dupilumab or its excipients: L-arginine hydrochloride, L-histidine, polysorbate 80, sodium acetate, acetic acid, sucrose, water for injections.
- Participants with active helminth and other parasitic infections.
- Patients infected with human immunodeficiency virus (HIV) (positive serology for HIV antibody).
- Patients testing positive for hepatitis B virus (HBV) or hepatitis C (HCV) infection.

*Assessments*

Subjects with a signed informed consent will be screened to ensure they meet all inclusion criteria and none of the exclusion criteria. The patients with an informed consent will undergo a screening which forms part of the inclusion phase. Patients will have their medical history taken and will undergo a physical exam by a physician. A blood sample will be taken during every visit, for assessment of the routinely laboratory assessments (same assessments as for dupilumab in AD). Female participants will undergo a urine pregnancy test and should be on an effective contraceptive during the treatment.

Patch testing must be performed within two years prior to screening, otherwise patients will undergo patch test procedure during screening. Patch testing will be performed with the European Baseline Series, fragrance series and cosmetic series which are applied on the back of the patient for 48 hours under occlusion. For patients with suspected contact allergy to other substances, we will also test with additional patch test series. Patch test readings will be performed according to the European Society of Contact Dermatitis (ESCD) guidelines on day 3 and day 7^3^.

After enrolment, the duration of the study is 16 weeks, involving a total of 5 study visits. Patients are instructed to make an unscheduled visit if the disease progresses during follow-up. **Table S1** gives an overview of all procedures during the course of the trial.

**Table S1.** Systematic overview of study procedures

| **Procedure** | **SCR** | **BL** | **V3W4** | **V4W8** | **V5W12** | **V6W16** | |
| --- | --- | --- | --- | --- | --- | --- | --- |
| Informed consent | X |  |  |  |  |  | |
| Medical/surgical history | X |  |  |  |  |  | |
| Inclusion/exclusion criteria | X |  |  |  |  |  | |
| Baseline characteristics |  | X |  |  |  |  | |
| Concomitant medications | X | X | X | X | X | X | |
| Adverse events |  |  | X | X | X | X | |
| Patch testing | X^1^ |  |  |  |  |  | |
| Injection instructions |  | X |  |  |  |  | |
| Providing medication |  | X | X | X | X |  | |
| Photographic Guide (PG) | X | X | X | X | X | X | |
| Physician Global Assessment (PGA) |  | X | X | X | X | X | |
| Hand Eczema Severity Index (HECSI) |  | X | X | X | X | X | |
| Quality of Life in Hand Eczema Questionnaire (QOLHEQ) |  | X | X | X | X | X | |
| Dermatology Life Quality Index (DLQI) |  | X | X | X | X | X | |
| Patient Global Assessment (PaGA) |  | X | X | X | X | X | |
| Work Productivity and Activity Impairment (WPAI) |  | X | X | X | X | X | |
| EuroQol Quality of Life 5-Dimension 5-Level (EQ-5D-5L) |  | X | X | X | X | X | |
| Photos |  | X | X | X | X | X | |
| Laboratory^2^ |  |  |  |  |  |  | |
| *- Complete blood count (CBC), Leukocyte count (WBC)* | X | X | X | X | X | X | |
| *- Thymus and activation regulated chemokine (TARC)* |  | X | X | X | X | X | |
| *- Total immunoglobulin (Ig) E, specific IgE for inhalant allergens* |  | X |  |  |  |  | |
| *- Creatinine, gamma-GT, ALAT* | X | X | X | X | X | X | |
| *- HIV screening* | X |  |  |  |  |  | |
| *- HBsAg, HBcAb, HepC* | X |  |  |  |  |  | |
| *- Pregnancy test for females of childbearing potential* | X |  |  |  |  |  | |
| *- Faeces test (only on indication^3^)* | X |  |  |  |  |  | |
| ^1^ Patch testing during Screening. Day 3 reading between Screening and Baseline (1 extra, short visit). Day 7 reading during Baseline.  ^2^ Withdrawal of blood before administering study drug injections  ^3^ In case of no visit to the tropics in history; no further action. In case of visit to the tropics in history; faeces PCR parasitic | | | | | | | |
| infections. Strongyloides and schistosomiasis serology in case of visit to a country where these diseases are endemic  (roughly Africa, South America, Asia and India). | | | | | | | |
|  | | | | | | |  |

At baseline data will be collected by prespecified medical charts for HE. These standardized forms include the following parameters: age at inclusion, sex, ethnicity, body-mass-index, atopy (allergic asthma was determined according to the Global Initiative for Asthma (GINA) guideline^4^ and allergic rhinitis according to the Allergic Rhinitis and its Impact on Asthma (ARIA) guideline)^5^, current and history of AD (both defined by U.K. Working Party criteria)^6,7^, age of onset of HE, morphologic classification of HE, work/activities, contact allergens, amount of contact with water, concomitant medication, treatment history (systemic therapy history and phototherapy history), intoxications (smoking). In case of smoking the amount of pack-years will be registered because these might be confounders. Pack years are calculated by multiplying the total years smoked with the average packs per day smoked over these years.

**
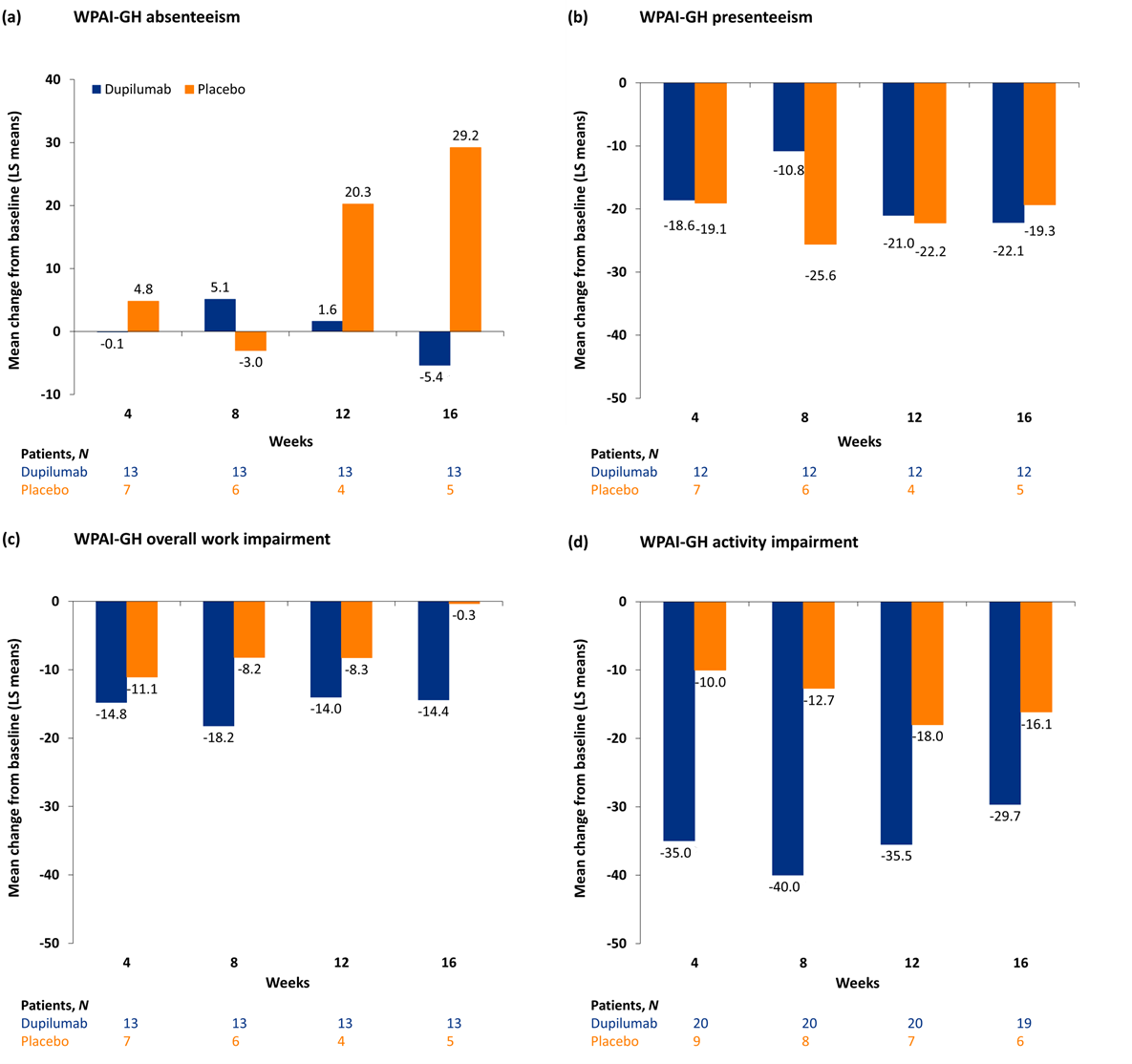
Supplemental figures and tables**

**Figure S1.** Work Productivity and Activity Impairment. Mean change from baseline in WPAI-GH scores for (a) absenteeism, (b) presenteeism), (c) overall work impairment and (d) activity impairment for dupilumab and placebo treatment groups at the following timepoints: week 4, week 8, week 12 and week 16. Data was analysed using a mixed model for repeated measurements. Missing data were imputed using the mixed model for repeated measurements predicted values. Higher scores indicate more impairment. Questions regarding absenteeism, presenteeism and overall work impairment were only answered by employed patients (13/20 patients in the dupilumab group, 7/9 patients in the placebo group). The question regarding activity impairment was answered by all patients. Patients who scored 0.0% on baseline scores for absenteeism, presenteeism, overall work impairment or activity impairment, were not included in this analysis. Numbers below the graph show the number of patients with non-missing values at all timepoints. *LS*, least square*; N*, number of patients; *WPAI-GH*, Work Productivity and Activity Impairment-General Health.

**Table S2. Overview of the allergen groups**

| **Allergen group** | **Composition** |
| --- | --- |
| Metals | Cobalt chloride, nickel sulfate, potassium dichromate, titanium (IV) oxalate hydrate |
| Preservatives | Chloromethylisothiazolinone, methyldibromo glutaronitrile, methylisothiazolinone,  2-methyl-4-isothiazolinone, 2-N-octyl-4-isothiazolin-3-one, paraben mix, sodium disulfit, sodium metabisulfite |
| Fragrances | Benzyl alcohol, fragrance mix I, hydroperoxides of linalool, myroxylon pereirae, peppermint oil, treemoss |
| Rubbers | Carba mix, benzisothiazolinone, black rubber mix, dibenzothiazyldisulfide, hydroquinone monobenzylether, mercapto mix, mercaptobenzothiazole, N-cyclohexyl-2-benzothiazylsulfenamide, N-cyclohexyl-N-phenyl-p-phenylenediamine, N-isopropyl-N-phenyl-4-phenyleendiamine, N,N'-diphenyl-p-phenylendiamine, tetraethylthiuramdisulfide, thiuram mix |
| Dyes/colours | *p*-Phenylenediamine, *p*-toluenediamine, textile dye mix, |
| Topicals | Amerchol L-101, benzoylperoxide, caïne mix, decyl glucoside, lanolin alcohols, propolis |
| Plastics | Epoxy resin, 2-hydroxyethyl methacrylate |
| Other | Colophony, p-tert-butylfenolformaldehyde resin |

**Table S3. Secondary efficacy outcomes: QOLHEQ subscales and EQ-5D-5L.**

| **Outcome** | **Week 4** | | **Week 16** | |
| --- | --- | --- | --- | --- |
|  | **Dupilumab**  **N = 20** | **Placebo**  **N = 9** | **Dupilumab**  **N = 20** | **Placebo**  **N = 9** |
| QOLHEQ – Symptoms |  |  |  |  |
| LSMean percentage change from baseline ±SE [95% CI] | -9.2 ±1.7 [-12.8;-5.6] | -4.0 ±2.6 [-9.3;1.3] | -16.6 ±2.8 [-22.4;-10.8] | -8.3 ±4.8 [-18.1;1.6] |
| LSMean change from baseline ±SE [95% CI] | -5.9 ±1.2 [-8.3;-3.5] | -2.4 ±1.7 [-6.0;1.1] | -8.4 ±1.2 [-10.9;-6.0] | -5.4 ±1.9 [-9.3;-1.6] |
| ≥6 points improvement from baseline, n/n (%) | 8/19 (42.1) | 1/9 (11.1) | 15/19 (78.9) | 3/9 (33.3) |
| QOLHEQ – Emotions |  |  |  |  |
| LSMean percentage change from baseline ±SE [95% CI] | -6.6 ±1.5 [-9.7;-3.5] | -6.0 ±2.3 [-10.6;-1.3] | -13.3 ±1.7 [-16.8;-9.7] | -9.4 ±2.9 [-15.3;-3.4] |
| LSMean change from baseline ±SE [95% CI] | -4.4 ±1.1 {-6.6;-2.1] | -3.8 ±1.6 [-7.1;-0.4] | -7.9 ±1.4 [-10.7;-5.0] | -6.4 ±2.2 [-10.9;-1.8] |
| ≥7 points improvement from baseline, n/n (%) | 3/15 (20.0) | 2/9 (22.2) | 10/15 (66.7) | 3/9 (33.3) |
| QOLHEQ – Functioning |  |  |  |  |
| LSMean percentage change from baseline ±SE [95% CI] | -8.4 ±2.1 [-12.7;-4.1] | -5.2 ±3.1 [-11.6;1.2] | -14.7 ±2.0 [-18.7;-10.6] | -10.8 ±3.2 [-17.2;-4.3] |
| LSMean change from baseline ±SE [95% CI] | -6.1 ±1.4 [-8.9;-3.3] | -3.0 ±2.1 [-7.2;1.2] | -9.2 ±1.5 [-12.2;-6.2] | -6.4 ±2.3 [-11.2;-1.7] |
| ≥8 points improvement from baseline, n/n (%) | 6/17 (35.3) | 1/8 (12.5) | 10/17 (58.8) | 3/8 (37.5) |
| QOLHEQ – Treatment and Prevention |  |  |  |  |
| LSMean percentage change from baseline ±SE [95% CI] | -4.3 ±1.8 [-8.0;-0.6] | -6.8 ±2.7 [-12.2;-1.3] | -11.9 ±1.6 [-15.1;-8.6] | -9.7 ±2.6 [-15.0;-4.4] |
| LSMean change from baseline ±SE [95% CI] | -3.6 ±1.1 [-5.8;-1.3] | -3.9 ±1.6 [-7.2;-0.6] | -6.8 ±1.1 [-9.0;-4.5] | -5.8 ±1.7 [-9.2;-2.3] |
| ≥5 points improvement from baseline, n/n (%) | 7/18 (38.9) | 3/9 (33.3) | 13/18 (72.2) | 5/9 (55.6) |
| EQ-5D-5L dimension |  |  |  |  |
| Self-care: reporting ‘no problems’, n/n (%) | 14/20 (70.0) | 6/9 (66.7) | 16/20 (80.0) | 5/9 (55.6) |
| Usual activities: reporting ‘no problems’, n/n (%) | 12/20 (60.0) | 4/9 (44.4) | 12/20 (60.0) | 4/9 (44.4) |
| Pain/discomfort: reporting ‘no problems’, n/n (%) | 4/20 (20.0) | 1/9 (11.1) | 9/20 (45.0) | 2/9 (22.2) |
| Anxiety/depression: reporting ‘no problems’, n/n (%) | 14/20 (70.0) | 5/9 (55.6) | 15/20 (75.0) | 6/9 (66.7) |

Binary endpoints were analysed using the Chi-square test or Likelihood-ratio test and missing values, e.g. data from patients after withdrawal from the study, were imputed using the last observation carried forward (LOCF). Continuous endpoints were analysed using a mixed model for repeated measurements and missing data were imputed using the mixed model for repeated measurements predicted values. Patients with <6 points on baseline QOLHEQ symptoms scores were excluded from the ≥6 points improvement on QOLHEQ symptoms analysis. Patients with <7 points on baseline QOLHEQ emotions scores were excluded from the ≥7 points improvement on QOLHEQ emotions analysis. Patients with <8 points on baseline QOLHEQ functioning scores were excluded from the ≥7 points improvement on QOLHEQ functioning analysis. Patients with <5 points on baseline QOLHEQ treatment and prevention scores were excluded from the ≥5 points improvement on QOLHEQ treatment and prevention analysis *CI,* confidence interval; *EQ-5D-5L*, generic five-dimension five-level EuroQoL scale; *LS*, least square; *n*, number of patients; *QOLHEQ*, Quality of Life in Hand Eczema Questionnaire; *SE*, standard error.

**Table S4. Sensitivity analysis for binary key outcomes**

| **Outcomes** | **Week 4** | | **Week 16** | |
| --- | --- | --- | --- | --- |
|  | **Dupilumab**  **N = 20** | **Placebo**  **N = 9** | **Dupilumab**  **N = 20** | **Placebo**  **N = 9** |
| HECSI |  |  |  |  |
| HECSI-50 response, n (%) | 16/20 (80.0) | 3/9 (33.3) | 19/20 (95.0) | 3/9 (33.3) |
| HECSI-75 response, n (%) | 9/20 (45.0) | 3/9 (33.3) | 19/20 (95.0) | 3/9 (33.3) |
| HECSI-90 response, n (%) | 3/20 (15.0) | 0/9 (0.0) | 14/20 (70.0) | 2/9 (22.2) |
| ≥ 41 points improvement from baseline, n(%) | 13/15 (86.7) | 3/8 (37.5) | 14/15 (93.3) | 2/8 (25.0) |
| PGA |  |  |  |  |
| Responders (‘clear’ or ‘almost clear’ and ≥2 points improvement), n (%) | 7/20 (35.0) | 2/9 (22.2) | 14/20 (70.0) | 3/9 (33.3) |
| Peak pruritus NRS |  |  |  |  |
| ≥4 points improvement from baseline, n (%) | 6/13 (46.2) | 2/8 (25.0) | 10/13 (76.9) | 2/8 (25.0) |
| QOLHEQ |  |  |  |  |
| Total: ≥22 points improvement from baseline, n (%) | 7/17 (41.2) | 1/9 (11.1) | 12/17 (70.6) | 3/9 (33.3) |

Binary endpoints were analysed using the Chi-square or Likelihood-ratio test and missing values were imputed using the last observation carried forward (LOCF). Patients were considered as non-responder after withdrawal from the study due to ineffectiveness and after use of rescue treatment. Patients with <4 points on baseline peak pruritus NRS were excluded from the ≥4 points improvement on peak pruritus NRS analysis. Patients with <22 points on baseline total QOLHEQ scores were excluded from the ≥22 points improvement on total QOLHEQ analysis. *HECSI*, Hand Eczema Severity Index; *LS*, least square; *n*, number of patients; *NRS*, Numerical Rating Scale; *PG*, photographic guide; *PGA*, Physician Global Assessment; *QOLHEQ*, Quality of Life in Hand Eczema Questionnaire.

| **Outcomes** | **Week 4** | | **Week 16** | |
| --- | --- | --- | --- | --- |
|  | **AD history N = 5** | **No AD history**  **N = 15** | **AD history**  **N = 5** | **No AD history**  **N = 15** |
| HECSI |  |  |  |  |
| HECSI-50 response, n (%) | 4/5 (80.0) | 12/15 (80.0) | 4/5 (80.0) | 15/15 (100.0) |
| HECSI-75 response , n (%) | 3/5 (60.0) | 6/15 (40.0) | 4/5 (80.0) | 15/15 (100.0) |
| HECSI-90 response , n (%) | 1/5 (20.0) | 2/15 (13.3) | 4/5 (80.0) | 10/15 (66.7) |
| PGA |  |  |  |  |
| Responders (‘clear’ or ‘almost clear’ and ≥2 points improvement), n (%) | 3/5 (60.0) | 11/15 (73.3) | 3/5 (60.0) | 11/15 (73.3) |
| Peak pruritus NRS |  |  |  |  |
| ≥4 points improvement from baseline, n (%) | 2/4 (50.0) | 4/9 (44.4) | 2/4 (50.0) | 8/9 (88.9.0) |
| QOLHEQ |  |  |  |  |
| Total: ≥22 points improvement from baseline, n (%) | 2/4 (50.0) | 3/11 (27.3) | 2/4 (50.0) | 8/11 (72.7) |

**Table S5. Sensitivity analysis for binary key outcomes: history of AD versus no history of AD**

Binary endpoints were analysed using the Chi-square or Likelihood-ratio test and missing values were imputed using the last observation carried forward (LOCF). Patients were considered as non-responder after withdrawal from the study due to ineffectiveness and after use of rescue treatment. Patients with <4 points on baseline peak pruritus NRS were excluded from the ≥4 points improvement on peak pruritus NRS analysis. Patients with <22 points on baseline total QOLHEQ scores were excluded from the ≥22 points improvement on total QOLHEQ analysis. *AD*, atopic dermatitis; *CI,* confidence interval*; HECSI*, Hand Eczema Severity Index; *LS*, least square; *n*, number of patients; *NRS*, Numerical Rating Scale; *PG*, photographic guide; *PGA*, Physician Global Assessment; *QOLHEQ*, Quality of Life in Hand Eczema Questionnaire.

**Table S6. Sensitivity analysis for binary key outcomes: dupilumab versus placebo in patients without history of AD**

| **Outcomes** | **Week 4** | | **Week 16** | |
| --- | --- | --- | --- | --- |
|  | **Dupilumab**  **N = 15** | **Placebo**  **N = 9** | **Dupilumab**  **N = 15** | **Placebo**  **N = 9** |
| HECSI |  |  |  |  |
| HECSI-50 response , n (%) | 12/15 (80.0) | 3/9 (33.3) | 15/15 (100.0) | 3/9 (33.3) |
| HECSI-75 response , n (%) | 6/15 (40.0) | 3/9 (33.3) | 15/15 (100.0) | 3/9 (33.3) |
| HECSI-90 response , n (%) | 2/15 (13.3) | 0/9 (0.0) | 10/15 (66.7) | 2/9 (22.2) |
| PGA |  |  |  |  |
| Responders (‘clear’ or ‘almost clear’ and ≥2 points improvement), n (%) | 5/15 (33.3) | 2/9 (22.2) | 11/15 (73.3) | 3/9 (33.3) |
| Peak pruritus NRS |  |  |  |  |
| ≥4 points improvement from baseline, n (%) | 4/9 (44.4) | 2/8 (25.0) | 8/9 (88.9.0) | 3/8 (37.5) |
| QOLHEQ |  |  |  |  |
| Total: ≥22 points improvement from baseline, n (%) | 3/11 (27.3) | 1/9 (11.1) | 8/11 (72.7) | 3/9 (33.3) |

Binary endpoints were analysed using the Chi-square or Likelihood-ratio test and missing values were imputed using the last observation carried forward (LOCF). Patients were considered as non-responder after withdrawal from the study due to ineffectiveness and after use of rescue treatment. Patients with <4 points on baseline peak pruritus NRS were excluded from the ≥4 points improvement on peak pruritus NRS analysis. Patients with <22 points on baseline total QOLHEQ scores were excluded from the ≥22 points improvement on total QOLHEQ analysis. *CI,* confidence interval; *HECSI*, Hand Eczema Severity Index; *LS*, least square; *n*, number of patients; *NRS*, Numerical Rating Scale; *PG*, photographic guide; *PGA*, Physician Global Assessment; *QOLHEQ*, Quality of Life in Hand Eczema Questionnaire.

**References**

1. Coenraads PJ, Van Der Walle H, Thestrup-Pedersen K, et al. Construction and validation of a photographic guide for assessing severity of chronic hand dermatitis. *Br J Dermatol*. Feb 2005;152(2):296-301. doi:10.1111/j.1365-2133.2004.06270.x

2. Menne T, Johansen JD, Sommerlund M, Veien NK, Danish Contact Dermatitis G. Hand eczema guidelines based on the Danish guidelines for the diagnosis and treatment of hand eczema. *Contact Dermatitis*. Jul 2011;65(1):3-12. doi:10.1111/j.1600-0536.2011.01915.x

3. Johansen JD, Aalto-Korte K, Agner T, et al. European Society of Contact Dermatitis guideline for diagnostic patch testing - recommendations on best practice. *Contact Dermatitis*. Oct 2015;73(4):195-221. doi:10.1111/cod.12432

4. Global Strategy for Asthma Management and Prevention. Global Initiative for Asthma. Accessed November 17, 2022. <https://ginasthma.org/>

5. Bousquet J, Khaltaev N, Cruz AA, et al. Allergic Rhinitis and its Impact on Asthma (ARIA) 2008 update (in collaboration with the World Health Organization, GA(2)LEN and AllerGen). *Allergy*. Apr 2008;63 Suppl 86:8-160. doi:10.1111/j.1398-9995.2007.01620.x

6. Williams HC, Burney PG, Pembroke AC, Hay RJ. The U.K. Working Party's Diagnostic Criteria for Atopic Dermatitis. III. Independent hospital validation. *Br J Dermatol*. Sep 1994;131(3):406-16. doi:10.1111/j.1365-2133.1994.tb08532.x

7. So How Do I Define Atopic Eczema? Appendix 2. University of Nottingham. Accessed November 17, 2022. <https://www.nottingham.ac.uk/~mzzfaq/dermatology/eczema/Section6-3Appendix2.html>
